# Incidence of Japanese Encephalitis and Acute Encephalitis Syndrome Hospitalizations in the Medium-Endemic Region in Central India

**DOI:** 10.1007/s44197-023-00110-7

**Published:** 2023-05-10

**Authors:** Babasaheb V. Tandale, Pravin S. Deshmukh, Shilpa J. Tomar, Rahul Narang, Mohiuddin S. Qazi, Padmaja Goteti Venkata, Manish Jain, Dipty Jain, Vijay Kumar Guduru, Jyoti Jain, Rajesh V. Gosavi, Chandra Sekhar Valupadas, Pradeep R. Deshmukh, Abhishek V. Raut, Uday W. Narlawar, Punam Kumari Jha, Vijay P. Bondre, Gajanan N. Sapkal, Rekha G. Damle, Poornima M. Khude, Abhimanyu K. Niswade, Manoj Talapalliwar, Pragati Rathod, Padmini Soujanya Balla, Pavan Kumar Muttineni, Kishore Kumar Kalepally Janakiram, Shekhar S. Rajderkar

**Affiliations:** 1 Epidemiology Group, ICMR-National Institute of Virology, 130/1, Sus Road, Pashan, Pune, Maharashtra 411021 India; 2 Mahatma Gandhi Institute of Medical Sciences, Sewagram, Wardha, Maharashtra India; 3 Government Medical College, Nagpur, Maharashtra India; 4 Kakatiya Medical College, Warangal, Telangana India; 5 Present Address: All India Institute of Medical Sciences, Bibinagar, Hyderabad India; 6 Present Address: All India Institute of Medical Sciences, Nagpur, Maharashtra India; 7 Government Medical College, Miraj, Maharashtra India

**Keywords:** Infectious encephalitis, Hospitalization rates, Incidence estimates, Impact assessment, Japanese encephalitis, Central India

## Abstract

**Background:**

We estimated the incidence of Japanese encephalitis (JE) and acute encephalitis syndrome (AES) following routine immunization with the live-attenuated SA 14-14-2 JE vaccine.

**Methods:**

We implemented enhanced surveillance of AES and JE hospitalizations in endemic districts in Maharashtra and Telangana States during 2015–2016 and 2018–2020. We estimated incidence and compared differences in the incidence of JE and AES between two states, and vaccinated and unvaccinated districts during two study periods. We also considered secondary data from public health services to understand long-term trends from 2007 to 2020.

**Results:**

The annual AES incidence rate of 2.25 cases per 100,000 children in Maharashtra during 2018–2020 was significantly lower than 3.36 cases per 100,000 children during 2015–2016. The six JE-vaccinated districts in Maharashtra had significantly lower incidence rates during 2018–2020 (2.03, 95% CI 1.73–2.37) than in 2015–16 (3.26, 2.86–3.70). In addition, the incidence of both JE and AES in two unvaccinated districts was higher than in the vaccinated districts in Maharashtra. Telangana had a lower incidence of both JE and AES than Maharashtra. The AES incidence rate of 0.95 (0.77–1.17) during 2018–2020 in Telangana was significantly lower than 1.67 (1.41–1.97) during 2015–2016.

**Conclusions:**

The annual incidence rate of Japanese encephalitis was < 1 case per 100,000 children. It indicated accelerated control of Japanese encephalitis after routine immunization. However, the annual incidence of acute encephalitis syndrome was still > 1 case per 100,000 children. It highlights the need for improving surveillance and evaluating the impacts of vaccination.

## Introduction

Japanese encephalitis (JE) is an important public health concern globally [1]. South-east Asia and the West Pacific region are the most endemic to JE, with 68,000 cases each year, mostly among children [1]. The actual incidence of JE is unknown. The estimates suggest yearly 14,000–20,000 fatal cases [2]. There is no specific treatment. However, vaccination is a safe and very effective preventive measure [3].

India is endemic to JE, with over 2500 cases and over 500 deaths per year [4]. The National Vector Borne Disease Control Programme (NVBDCP) reports thousands of cases of acute encephalitis syndrome (AES) and also JE every year in India [5]. The JE cases are decreasing recently due to awareness and vaccination [6]. The AES and JE cases are reported year-round in Telangana and Maharashtra States in the central region of India among children [7] and also adults [8].

The JE vaccination using the live-attenuated 14-14-2 JE vaccine was first introduced in priority endemic districts in 2006 and thereafter in a phased manner as a single 0.5 ml subcutaneous dose during mass vaccination campaigns among 1–15 years children [9]. A single dose of the JE vaccine was later included in the routine immunization schedule for children aged 16–24 months and was implemented until 2013. Later, since 2013, two doses were implemented in routine immunization schedules in priority endemic districts implemented with mass vaccination campaigns. The first dose was recommended at 9–12 months of age and the second dose at 16–24 months was co-administered with the Measles-Rubella vaccine [10].

In India, AES is primarily caused by the JE virus, mostly among children. Despite JE vaccination implementation in routine immunization schedules following mass vaccination campaigns, JE disease continues to occur [7, 11], though not with epidemic potential, but with a significantly high case fatality rate and lifelong sequelae among survivors, which are lacking the much-needed attention and efforts requires for awareness, support and care [8].

There are no studies estimating incidence rates of encephalitis in medium-endemic Central India following the implementation of JE vaccination. Hence, we estimated the AES and JE incidence rates in Telangana and Maharashtra States in post-JE vaccine implementation periods for assessing the impacts of JE vaccination on the incidence of JE and AES.

## Methods

### Study Design

We estimated incidence based on two surveillance studies using enhanced surveillance of AES and JE hospitalizations in endemic districts of Maharashtra and Telangana, implemented during 2015–2016 and 2018–2020. In addition, we used secondary data and reports of JE and AES cases reported by public health departments for understanding long-term trends during 2007–2020 following the implementation of JE vaccination among children.

### Setting

The sentinel hospital-based enhanced surveillance was undertaken in endemic areas as a multi-centric study in 8 districts of eastern Maharashtra and 16 districts of northern Telangana.

### Study Population and Area

We considered surveillance and investigation of hospitalizations across all age groups during 2018–2020, whereas only children < 15 years of age were studied during 2015–2016. The cases for these studies were the patients hospitalized from all six districts of the Nagpur division of health services and adjoining Yavatmal and Amravati districts from the Akola division of health services in Maharashtra State, along with 16 districts in the Warangal division of health services in Telangana State.

### Study Procedures

The Institutional Ethics Committees approved the study protocol at the reference laboratory and collaborating hospitals. We sought written informed consent from parents or guardians before the enrollment of patients in the study.

### Surveillance and Case Definitions

We actively searched all neurological hospitalizations in pediatric and medicine wards for the verification of clinical features using the case definition of AES implemented by the NVBDCP, followed by the specific criteria for study eligibility after considering the differential diagnosis by treating physicians and confirmed diagnoses based on initial clinical and laboratory evaluations [7]. We utilized serum and cerebrospinal fluid (CSF) testing results for the estimation of incidence based on JE-confirmed diagnosis [7]. We used these data for incidence rate estimations for two study periods of surveillance.

We also estimated the annual incidence rates of AES and JE based on the public health reported data (secondary data) along with utilizing the surveillance data (primary data) collected during the year 2015–2016 and 2018–2020. We used person-year as the denominator for the estimation of incidence rates. We also attempted a comparison of incidence estimates based on public health reports and surveillance studies. The annual incidence rate was expressed as the number of JE and AES cases per 100,000 population per year. The population of Maharashtra and Telangana States and included districts was obtained from the Census of India.

### Statistical Analysis

We used MS Excel for data compilation and management and online statistical software for person-time estimation. We summarized the incidence rates with 95% confidence intervals for each year, for study districts, along with vaccinated and unvaccinated study districts and also for comparisons between children and adults. The trends of AES and the JE incidence in two states were analyzed using interrupted time-series analysis. We considered a *p* value of < 0.05 as significant for comparisons of incidence rates.

## Results

We present the estimates of the annual incidence rates of AES and JE at the state level based on the secondary data reported by the public health departments for understanding the long-term trends after the two-dose routine JE vaccination (2014–2020) in comparison to the period of two-dose implementation (2007–2013). In addition, we present the incidence rate estimates for the study districts based on the primary data from surveillance research studies in two different 2-year study periods.

A. Incidence rate estimates based on secondary data reported by public health departments.
1. Incidence rate estimates of AES and JE at the state level in Maharashtra and Telangana.

**Fig. 1:**
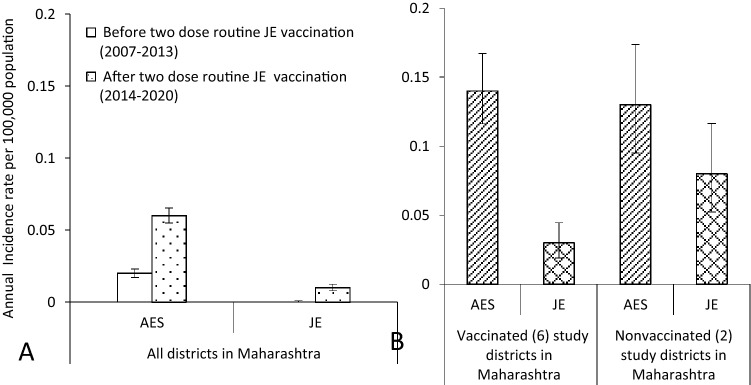
Incidence rate estimates of acute encephalitis syndrome (AES) and Japanese encephalitis (JE) cases per 100,000 population before and after implementation of two doses of JE vaccine in routine immunization schedule in Maharashtra based on public health reported cases. **a** Incidence estimates at the state level. **b** Incidence estimates in vaccinated and nonvaccinated study districts. Error bars (whiskers) indicate 95% confidence levels for estimates

In Maharashtra, both AES and JE showed an increase in incidence at the state level after two-dose vaccination (Fig. 1a). After two-dose implementation, a declining trend of AES was noted in both states, though it was nonsignificant.

Based on the state-level data reported by public health departments, the annual incidence rate of both AES and JE was < 1 case per 100,000 population for Maharashtra State before the JE vaccination implementation period (2007–2013) and also after two-dose JE vaccine implementation (2014–2020) (Fig. 1a). The JE and AES incidence in the pre-vaccination implementation period was significantly higher in Telangana as compared to Maharashtra (*p* < 0.001). However, during the post-vaccine implementation period, the annual incidence rate per 100,000 population for AES in Telangana was 0.22 (95% CI 0.21–0.24) and 0.03 (95% CI 0.02–0.03) for JE.

2. Incidence rate estimates in study districts of Maharashtra as per the JE vaccination status

The annual AES incidence rate of 0.14 (95% CI 0.11–0.16) cases per 100,000 population in six vaccinated districts during 2014–2020 (Fig. 1b) was significantly lower as compared to before the vaccination implementation period [0.24 (95% CI 0.20–0.28)] (*p* < 0.001). The annual JE incidence rate during 2014–2020 was slightly higher in nonvaccinated districts than in vaccinated districts in Maharashtra (Fig. 1b).

B. Incidence rate estimates based on primary data from surveillance research studies.
1. Incidence rate estimates of JE and AES among children during 2015–2016 and 2018–2020.

**Fig. 2:**
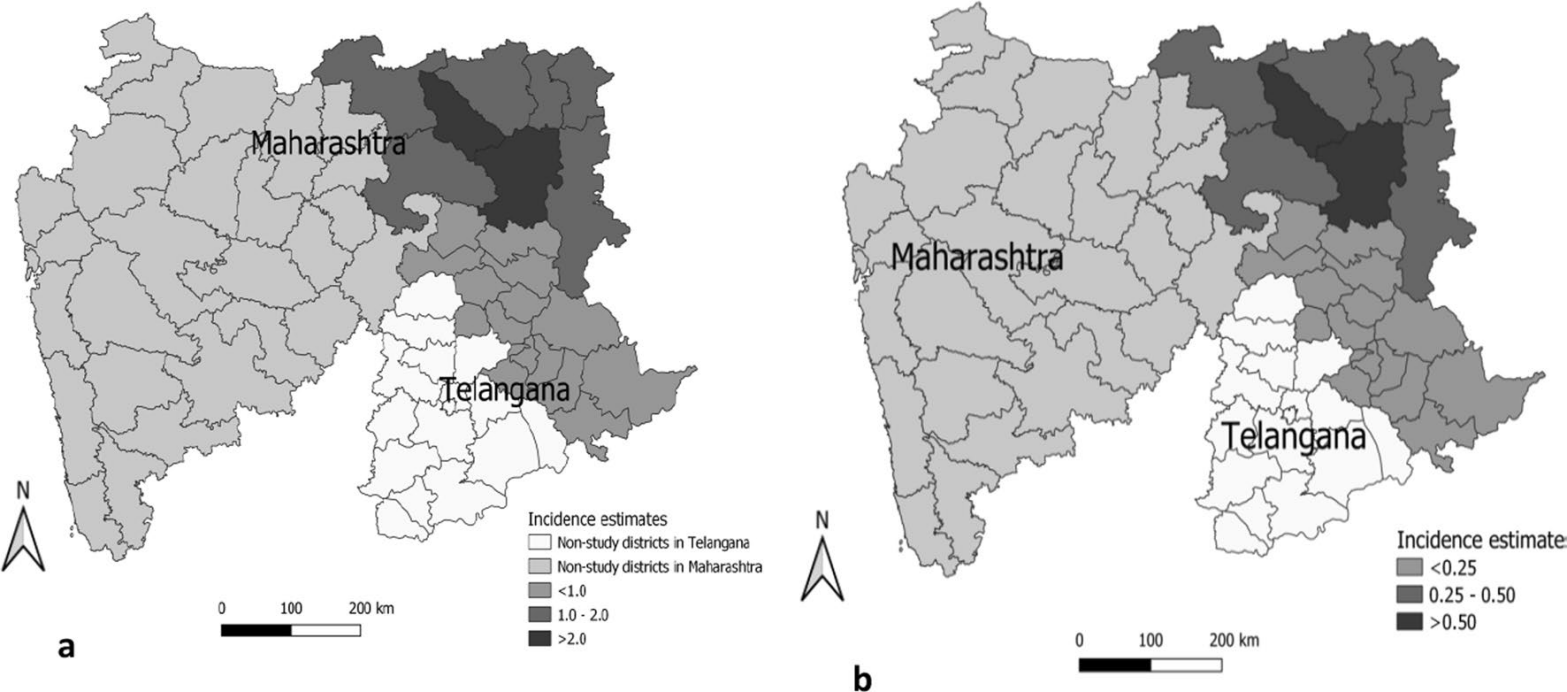
Incidence rate estimates per 100,000 children per year in study districts in Maharashtra and Telangana as per the JE vaccination status during 2018–2020. **a** Acute Encephalitis Syndrome (AES). **b** Japanese Encephalitis (JE)

The incidence rate estimates of AES in study districts in Maharashtra during 2018–2020 (Fig. 2a) were lower as compared to 2015–2016 (data not shown). However, the incidence rate estimate of JE in study districts in Maharashtra was more than two times higher during 2018–2020 (Fig. 2b) as compared to 2015–2016 (data not shown). In Telangana also, the incidence rate of AES in study districts was lower during 2018–2020 (Fig. 1a) as compared to 2015–2016 (data not shown). The incidence rate of JE was slightly lower in study districts of Telangana during 2018–2020 (Fig. 1b) as compared to 2015–2016 (data not shown).

**Fig. 3:**
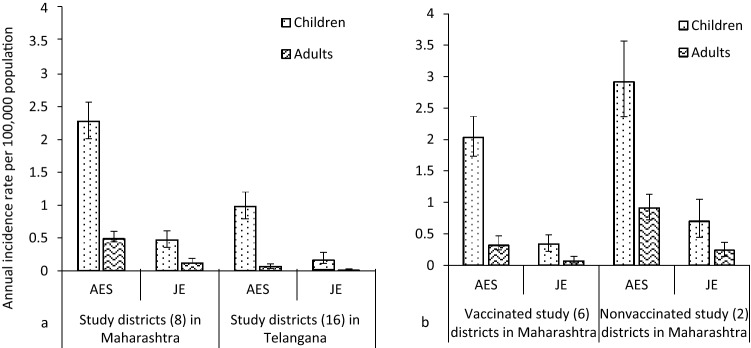
Incidence rate estimates of acute encephalitis syndrome (AES) and Japanese encephalitis (JE) cases per 100,000 population among children and adults during 2015–2016 and 2018–2020 after the implementation of routine immunization of infants with two doses of the live-attenuated SA 14-14-2 JE vaccine. **a** Study districts in Maharashtra and Telangana. **b** Vaccinated and nonvaccinated study districts in Maharashtra

The overall incidence rate of both AES and JE in study districts in Maharashtra was more than two times higher as compared to Telangana (Fig. 3a). Incidence rates of AES in nonvaccinated study districts in Maharashtra were significantly higher during 2018–2020 as compared to 2015–2016. The incidence rate of AES in nonvaccinated districts in Maharashtra during 2018–2020 was over two cases per 100,000 population, which was higher than in vaccinated districts with the increase seen specifically in JE incidence in both nonvaccinated and vaccinated study districts in Maharashtra (Fig. 3b).

2. Incidence rate estimates of JE and AES among both children and adults during 2018–2020.

**Fig. 4:**
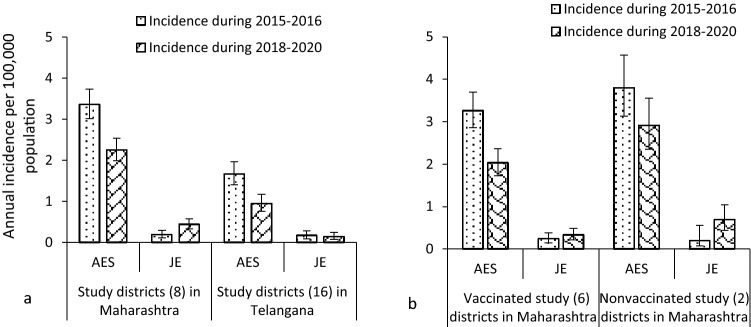
Incidence rate estimates of acute encephalitis syndrome (AES) and Japanese encephalitis (JE) cases per 100,000 population among both children and adults after the implementation of routine immunization with two doses of the live-attenuated SA 14-14-2 JE vaccine among infants during 2018–2020. **a** Incidence estimates for the study districts in Maharashtra and Telangana. **b** Incidence estimates for the JE-vaccinated and nonvaccinated study districts in Maharashtra

The incidence rate estimates of AES and JE were significantly lower among both children and adults in both states during 2018–2020 (Fig. 4a). The AES incidence rate among children in Maharashtra was more than two cases per 100,000 population as compared to Telangana having < 1 case per 100,000 population. In Maharashtra, incidence rate estimates of both AES and JE were lower in vaccinated study districts than in nonvaccinated study districts (Fig. 4b).

In summary, incidence rate estimates based on the public health reported secondary data showed achievement of accelerated control of JE with less than one case of JE and also AES per 100,000 population per year. Telangana reported lower annual JE and AES incidence rates than Maharashtra. The JE incidence rate estimates in nonvaccinated districts were slightly higher as compared to vaccinated districts in Maharashtra State.

## Discussion

Although the JE virus is a prominent cause of AES in India with an enormous neurological disease burden, the incidence estimates of JE and also AES are not documented in India, though cases and outbreaks of JE are continuing to be reported in different regions [3, 12, 13]. JE is a major public health problem among children in medium-endemic Central India [7]. The national estimate [14] of the incidence rate of JE was 1.14 cases during 2007–2011 and 1.08 cases per 1,000,000 population during 2013–2021 based solely on reported secondary public health data. However, the incidence rate estimates for different endemic regions in India are not available and also not reliable as they are not based on properly planned surveillance data and only used secondary reported public health data [1].

In our study, the incidence rate estimates of JE and AES in both Maharashtra and Telangana States based on public health reported secondary data indicated the achievement of accelerated control of both JE and AES. However, a slight increase in the incidence of both JE and AES in Maharashtra was seen in the post-JE vaccine two-dose-schedule implementation period during 2014–2020 in comparison to the earlier period using a single dose implemented during 2007–2013. In addition, the JE incidence rate was slightly higher in nonvaccinated study districts than in vaccinated study districts in Maharashtra. Telangana state had a lower incidence of AES and also JE in both children and adults than Maharashtra. It may be due to all study districts in Telangana having implemented JE vaccination as against six of eight study districts in Maharashtra. However, in our enhanced surveillance studies in both states, annual AES incidence among children was > 2 cases per 100,000 population, although JE incidence was within accelerated control levels.

Earlier incidence estimations were made before 2013, the period when the JE vaccination implementation in the routine immunization schedule was not implemented. India has been divided into low, medium and high JE endemicity based on the reported JE cases [1]. Very few studies have reported the incidence estimates [15], and those reported are mostly from high-endemic areas. In addition, the incidence rate estimates were imprecise, mostly based only on secondary reported public health data.

At the national level, only one study reported incidence estimates [15], along with the forecasted incidence rate in India to be 0.49 (95% CI 0.19–1.06) for 2012 and 0.42 (95% CI 0.15–0.97) for 2013. In high-endemic regions, the incidence rate of AES from 1978 to 2011 was 0.42 cases per 100,000 population and during 1993–2000, it was one per 100,000 population. During 2008–2012, in the Gorakhpur division [16], the average annual incidence of JE was 1.1–1.4 cases per 100,000 population, with the incidence decreasing since 2010, from 1.9 per 100,000 in 2010 to 0.5 per 100,000 in 2012. In 2011, the estimated AES incidence of all ages was 20.2 per 100,000 population. The incidence among children aged 0–6 years was 7.97 (6.87–9.25) in Kushinagar in Uttar Pradesh [17]. In Bihar [18], the annual incidence rate of AES was 4.7–25.0 and 0.55–1.78 of JE cases per 100 000 population during 2009–2014. Recently, the JE incidence estimate of 10.5 cases per 100,000 in the pre-vaccination period among adults in Assam during 2009–2018 has decreased to 5.7 per 100,000 in the post-vaccination period [19]. In Assam, from January 2011 to December 2013, JE incidence estimates were 2.9 cases per 100,000 children per year after the live-attenuated SA 14-14-2 JE vaccine was implemented among adults in endemic districts [20].

In summary, AES and JE incidence significantly declined among children in both Maharashtra and Telangana States. We observed higher AES and JE incidence in both children and adults in nonvaccinated districts than in vaccinated districts. JE incidence is increasing in adults indicating the need to continue surveillance enhancement for tracking adult JE and AES cases to help evaluation of the need for consideration of adult JE vaccination.

### Limitations

We could not estimate the JE and AES incidence rates at the district levels because of the very low number of JE and AES cases reported during the period of interest. In addition, we could only estimate the incidence rates of JE and AES during the post-vaccine implementation period as our both surveillance studies were implemented after 2014. We may have underestimated the incidence rates as we used only the hospital-based surveillance data, although there are very less chances of cases being missed due to the disease severity of encephalitis-like illness.

## Conclusions

Japanese encephalitis continued to occur in the medium-endemic region of Central India, although at lower levels as compared to high-endemic regions. The incidence of acute encephalitis syndrome in the medium-endemic region in Central India is still high after the implementation of childhood Japanese encephalitis vaccination in routine immunization schedules. The national programme activities including surveillance, management and vaccination need to positively consider strengthening efforts for monitoring and consider evaluating the need for the expansion of vaccination to new areas that reported cases recently.

## Data Availability

The authors have the data for the manuscript. It may be considered for sharing at the request of the readers.

## Abbreviations

AES: Acute encephalitis syndrome
CSF: Cerebrospinal fluid
ICMR: Indian Council of Medical Research
JE: Japanese encephalitis
NVBDCP: National Vector Borne Disease Control Programme
